# Digital pathology for the routine diagnosis of renal diseases: a standard model

**DOI:** 10.1007/s40620-020-00805-1

**Published:** 2020-07-18

**Authors:** Vincenzo L’Imperio, Virginia Brambilla, Giorgio Cazzaniga, Franco Ferrario, Manuela Nebuloni, Fabio Pagni

**Affiliations:** 1 Department of Medicine and Surgery, Pathology, San Gerardo Hospital, University of Milano-Bicocca, Monza, Italy; 2 Pathology Unit, ASST Sacco-Fatebenefratelli, University of Milan, Milan, Italy

**Keywords:** Digital pathology, Renal pathology, Telepathology, Renal biopsy

## Abstract

Whole-slide imaging and virtual microscopy are useful tools implemented in the routine pathology workflow in the last 10 years, allowing primary diagnosis or second-opinions (telepathology) and demonstrating a substantial role in multidisciplinary meetings and education. The regulatory approval of this technology led to the progressive digitalization of routine pathological practice. Previous experiences on renal biopsies stressed the need to create integrate networks to share cases for diagnostic and research purposes. In the current paper, we described a virtual lab studying the routine renal biopsies that have been collected from 14 different Italian Nephrology centers between January 2014 and December 2019. For each case, light microscopy (LM) and immunofluorescence (IF) have been processed, analysed and scanned. Additional pictures (eg. electron micrographs) along with the final encrypted report were uploaded on the web-based platform. The number and type of specimens processed for every technique, the provisional and final diagnosis, and the turnaround-time (TAT) have been recorded. Among 826 cases, 4.5% were second opinion biopsies and only 4% were suboptimal/inadequate for the diagnosis. Transmission electron microscopy (TEM) has been performed on 41% of cases, in 22% changing the final diagnosis, in the remaining 78% contributed to the better definition of the disease. For light microscopy and IF the median TAT was of 2 working days, with only 8.6% with a TAT longer than 5 days. For TEM, the average TAT was 26 days (IQR 6–64). In summary, we systematically reviewed the 6-years long nephropathological experience of an Italian renal pathology service, where digital pathology is a definitive standard of care for the routine diagnosis of glomerulonephritides.

## Introduction

Digital pathology consists of a complex group of technological sources (whole slide imaging, WSI, virtual microscopy, VM, image analysis and derived complex processes such as neural networks) that are revolutionizing the routine workflow [1]. In the last decade the application of such tools for second-opinion purposes on definitive or frozen-sections (telepathology) has been definitely consolidated [2]. Their application for multidisciplinary team (MDT) meetings and educational tasks are being progressively implemented, mutuating from the experience of digital radiology [3]. These examples culminated in the full conversion of entire pathology departments to the digital slides [4]; this was a process facilitated by the Food and Drugs Administration (FDA) approval for the clinical employment of “the digital” in the routine pathological practice. This paradigm shift, however, requires an optimization of the specimen processing phases (pre, post and analytical) such as an appropriate laboratory information system (LIS) to ensure the adequate patient identification and error reduction [5]. In renal pathology, the integration of light microscopy (LM) with (time-sensitive) immunofluorescence (IF) and transmission electron microscopy (TEM) is a required aim to develop a reliable and completely dynamic reproduction of the original specimens. Here is discussed the 6-years experience of an Italian renal pathology service in which telepathology is a definitive standard of care for the routine management of glomerulonephritides.

## Materials and methods

Routine renal biopsies were physically sent for diagnosis or second opinion consultation at the Pathology Unit of ASST Monza, Italy from 14 different Italian Nephrology centers (9 from the North, 1 from the Center and 4 from the South of Italy). Upon arrival at the pathology department, the specimens have been processed following a standardized protocol [6, 7]. Depending on the distance of the referral center, tissue specimens for first diagnosis have been sent either (i) divided directly by the nephrologists at the bedside in three different appropriate media for each analysis technique (formalin for LM, preservative medium such as Michel’s or saline solution for IF and glutaraldehyde for TEM) or (ii) directly fixed in formalin without subdivision of the sample. For second opinion purposes formalin fixed paraffin embedded (FFPE) blocks were received and processed as follows. For light microscopy slides were stained with routine histochemical methods hematoxylin and eosin -H&E-, periodic acid–Schiff reaction -PAS-, silver methenamine and trichrome stain. Direct or indirect IF has been performed for each case depending on the available material (fresh vs FFPE), as previously described [6, 7]; biopsies were routinely tested for immunoglobulins (primarily IgG, IgM and IgA), complement components (C3 and C1q), fibrinogen, and kappa and lambda light chains. The ultrastructural analysis (TEM) has been performed at the Pathology Unit, ASST Sacco Fatebenefratelli, University Milan. The samples were sent after glutaraldehyde fixation or as FFPE blocks (in cases without dedicated specimen) and processed in an interval of time of 3–5 working days. The selection of cases requiring TEM has been made following the judgement of the renal pathologist and the referral nephrologist for each case. Common reasons that led to the ultrastructural analysis were: (i) inconsistency with clinical data and LM/IF findings, (ii) confirmation of immune complex deposits, (iii) characterization of structure and distribution of the deposits, (iv) quantification of the podocyte damage, (v) measure of the glomerular basement membrane (GBM) thickness and (vi) LM/IF inadequate samples. On the base of whether electron microscopy contributed to the final diagnosis, cases have been assigned to three main categories, borrowing the definitions previously provided by Mark Haas [8], as follows:

- *Essential* “Electron microscopy was needed to make the primary final diagnosis either changing the preliminary diagnosis or resolving a differential diagnosis in cases where a firm preliminary diagnosis could not be made.”
- *Informative* “The ultrastructural findings did not alter the preliminary diagnosis and were not essential to making the primary final diagnosis. However, the ultrastructural findings did provide important information confirming/strengthening this primary diagnosis and/or provided clinically relevant insight into the patient’s historical data, light microscopic findings, and/or immunofluorescence findings related to the primary diagnosis.”
- *Not relevant* “Electron microscopy resulted in no change in the preliminary diagnosis, was not needed to confirm this diagnosis, and did not supply other clinically pertinent information related to the primary final diagnosis.”

For the purpose of the study a final list from January 2014 to December 2019 of 826 complete cases was included. The retrospective analysis of this series allowed the collection of the number and type of specimens processed for every technique (eg fresh frozen, FFPE, glutaraldehyde fixed), the provisional diagnosis after LM and IF, the definitive diagnosis after TEM, and the turnaround-time (TAT) after LM and IF and after TEM. Numerical continuous variables are reported as median and interquartile range (IQR).

### Digital microscopy workflow

**Fig. 1:**
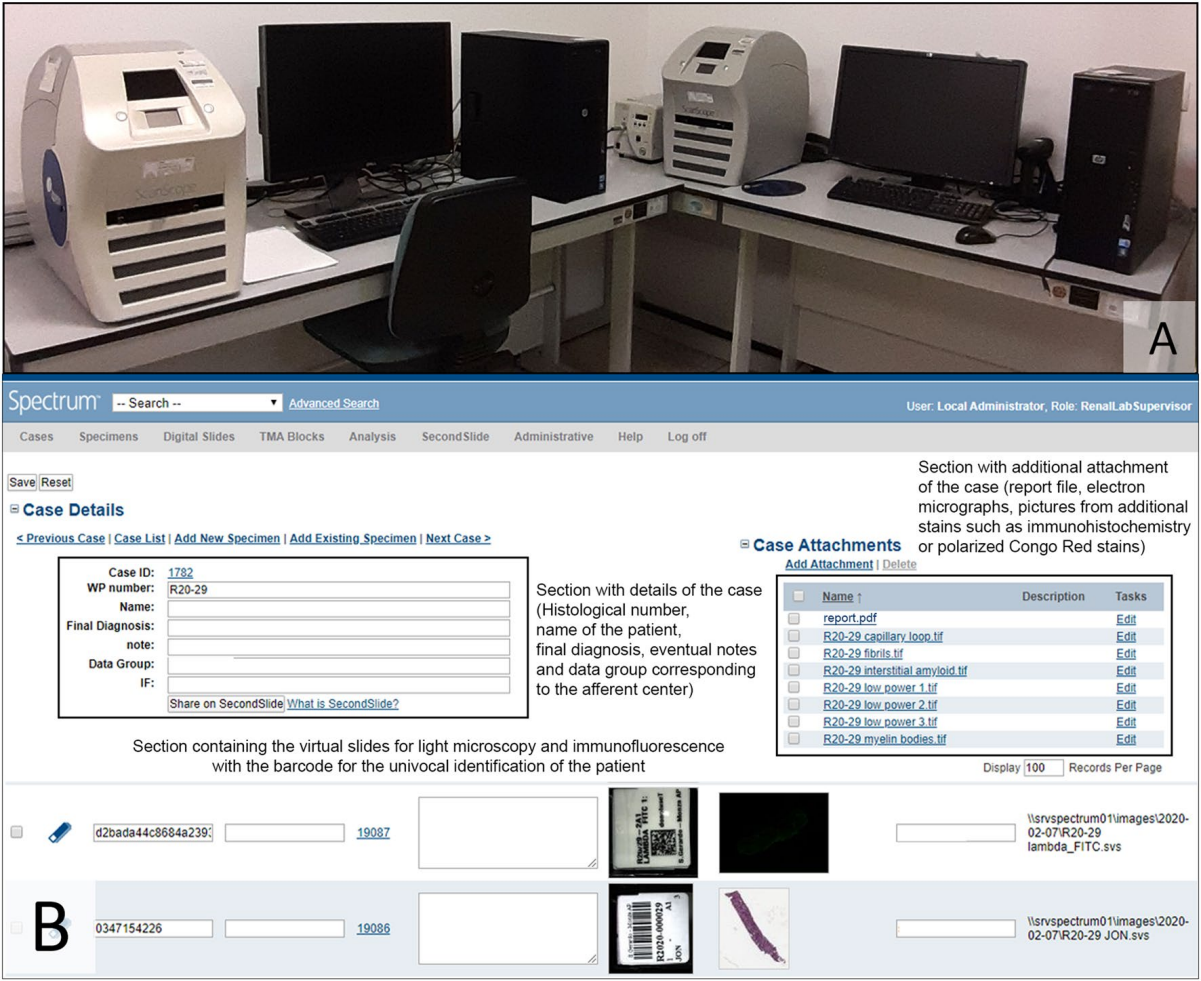
**a** The instrumentation employed in the facility of ASST Monza. On the left Aperio CS2 device for light microscopy and Aperio ScanScope FL for immunofluorescence on the right. **b** The single case as it is displayed in the platform Spectrum for every afferent center. On the upper left black box is the section with the details of the case (histological progressive number, name of the patient, final diagnosis, eventual notes and the data group, corresponding to each afferent center). On the upper right black box is the section dedicated to the additional attachments, such as the final report, electron micrographs and pictures captured from ancillary techniques (immunohistochemistry or Congo Red stain). On the bottom of the picture the rows with virtual slides of the case, with both light microscopy and immunofluorescence, associated with the appropriate barcode to ensure the correct identification of the patient

Routinely, biopsies were scanned using Aperio CS2 device for LM and Aperio ScanScope FL for IF (Leica Biosystem, Fig. 1a). For IF all the positive slides have been captured with a static color microscope camera (Leica DFC425C, Leica Biosystem, Illinois, USA) mounted on a fluorescence microscope (Zeiss Axio Lab A1, Jena, Germany) and relative pictures saved on a shared static server of the ASST Monza. Positive slides for each case have been scanned either (i) using the focus and exposition time automatically set by the scanner and subsequently (ii) manually adjusting the parameters on the base of those used for the static camera acquisition process. Once obtained, digital slides were then imported in the Spectrum platform and assigned to the appropriate case through the employment of a barcode. Additional pictures deriving from either special histochemistry techniques (eg Congo Red birefringence) or electron micrographs were uploaded on the specific page of the case under an appropriate section (Case Attachments). Once the final report was generated by the local system, an encrypted pdf file generated by the LIS was created and uploaded on the same “Case Attachments” section to be easily retrievable by the referral clinician (Fig. 1b).

## Results

The study included 826 cases, 4.5% (37) of which were second opinion biopsies sent as FFPE blocks. IF has been performed on fresh frozen material in 70% (580) of cases, whereas paraffin material has been used for the other 30% (246). Electron microscopy has been performed on 41% (340) of cases, 84% (284) of which with dedicated glutaraldehyde fixed specimen and 16% (56) of which after retrieval from FFPE blocks. In 5% (18) of cases TEM was requested for inconsistency with clinical data and LM/IF findings, in 21% (72) for the detection/exclusion of immune complex deposits, in 38% (130) for the characterization of structure and distribution of the deposits, in 25% (86) for the quantification of the podocyte damage (foot process effacement), in 5% (17) for the measurement of the GBM thickness and in 5% (17) because of LM/IF inadequate samples. The execution of ultrastructural analysis has been essential in 22% (75) of cases, informative in the remaining 48% (163) and not relevant in a further 30% (102). The role of electron microscopy has been considered essential in all cases with minimal change disease (MCD, *n* = 57), in the cases with focal segmental glomerulosclerosis (FSGS, *n* = 29) in which has been performed, as well as in Fabry nephropathy (*n* = 6), Alport disease/thin glomerular basement membrane (*n* = 7), fibrillary (*n* = 3) and immunotactoid glomerulonephritis (*n* = 1). The technique demonstrated to be informative in many cases (75%, *n* = 40) with membranous nephropathy (MN) as well as with lupus nephritis (LN) in which has been performed (90%, *n* = 23). In the setting of a preliminary diagnosis of C3 glomerulopathy, TEM allowed to sub-classify the cases as C3 glomerulonephritis (*n* = 9) or as dense deposits disease (*n* = 2). Its role has been much more limited or not relevant with entities well definable through LM and IF, such as in the biopsies with IgA nephropathy in which has been performed (*n* = 29), adding significant informations only in a minority of these cases (34%, *n* = 10). Finally, in diabetic nephropathy (DN) and arterionephrosclerosis (ANS) ultrastructural analysis only rarely added useful informations, leading to an unmutated final diagnosis in 80% (*n* = 8) and 83% (*n* = 19) of the cases in which has been performed, respectively.

**Fig. 2:**
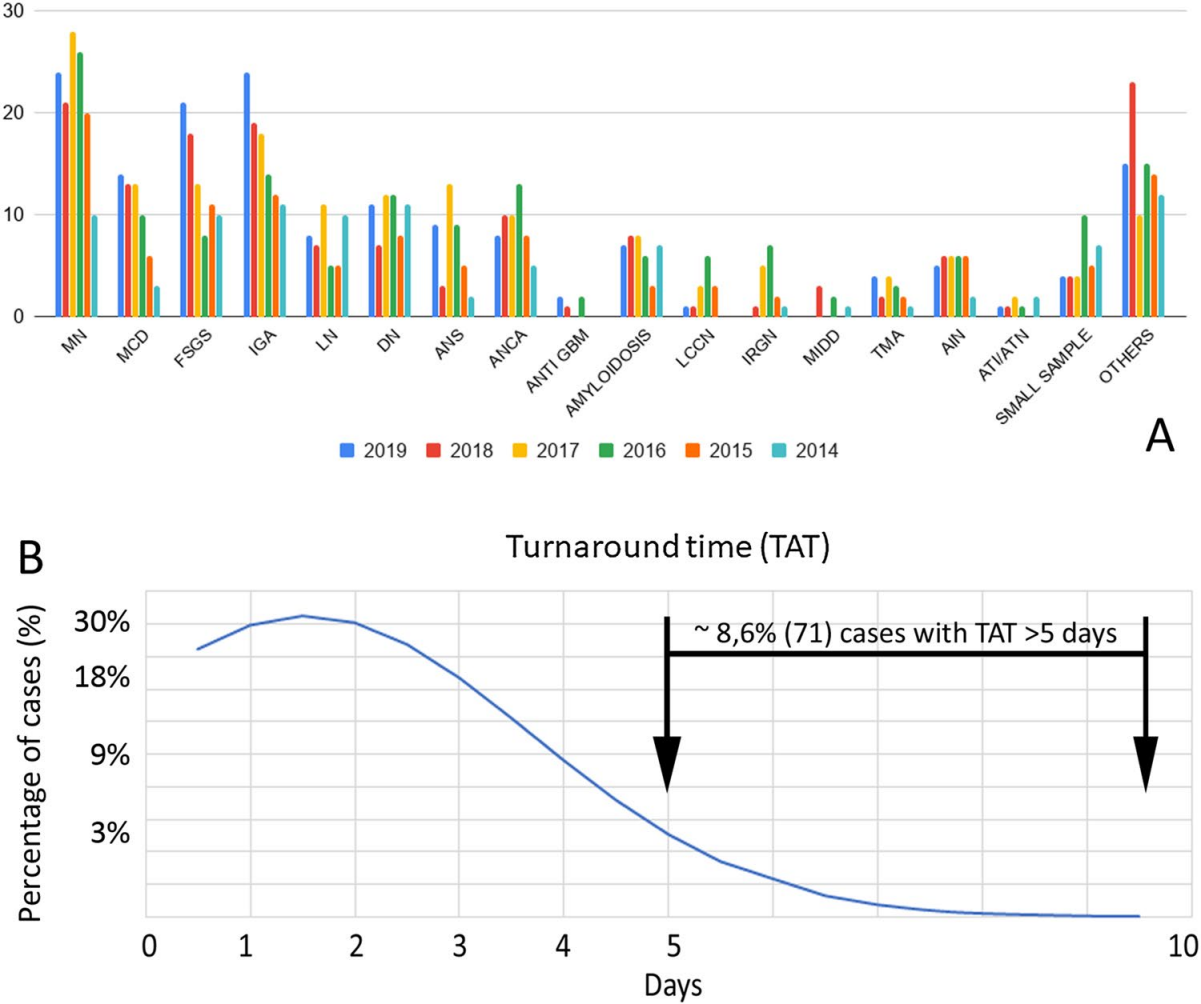
**a** Distribution and frequency of the final diagnosis per year. MN membranous nephropathy, MCD minimal change disease, FSGS focal segmental glomerulosclerosis, IgA IgA nephropathy, LN lupus nephritis, DN diabetic nephropathy, ANS arterionephrosclerosis, ANCA pauci immune crescentic glomerulonephritis, ANTI GBM Anti-GBM glomerulonephritis, LCCN light chain cast nephropathy, IRGN infection related glomerulonephritis, MIDD monoclonal immunoglobulin deposition disease, TMA thrombotic microangiopathy, AIN acute interstitial nephritis; OTHERS: some of the other rare diagnosis were represented by acute pyelonephritis, Alport syndrome and thin basement membrane lesion, cryoglobulinemic glomerulonephritis, fibrillary glomerulonephritis, immunotactoid glomerulonephritis, renal lymphoma, atheroembolic disease, IgA vasculitis and much rarer diseases. **b** The distribution of cases on the base of the TAT. The majority of them were managed within 2–3 working days (median 2, IQR 1–3) and only 8.6% (71) cases had a TAT > 5 days (half of them, 37, coming from the South of Italy)

Overall, the most frequent final diagnosis of the series was represented by MN (16%) followed by IgA nephropathy (12%), FSGS (10%,), MCD (7%), pauci immune crescentic glomerulonephritis (7%), DN (7%), LN (6%), ANS (5%), amyloidosis (5%) and tubulointerstitial nephritis (4%). Only 4% of cases (*n* = 34) were suboptimal/inadequate for the diagnosis (eg. absence or low number of glomeruli for all the three technique, not allowing a definitive diagnosis). The remaining 18% (*n* = 153) were characterized by rarer forms of renal diseases. The incidence of each disease for the whole 6-years period is quite unmutated if the single year frequency is considered, as depicted in Fig. 2a, even if a progressive increase in the number of cases collected from 2014 to 2019 can be observed (from 95 to 158 renal biopsies). For LM and IF the median TAT was of 2 working days (IQR of 1–3). Less than 10% of cases (8.6% of cases), half of which from regions of the South of Italy (Fig. 2b), had a TAT longer than 5 working days. The median time to the full report, comprehensive of TEM in cases needing ultrastructural analysis, was 26 working days (IQR 6–64). The time required to access to the virtual slide on the platform for both the pathologist and the referral nephrologist is of 30 s in average.

## Discussion

The gradual increase in complexity of glomerulonephritides classifications led to the creation of the nephropathologist figure, with consequent proposal to centralize renal biopsies from small peripheral centers (spoke) to bigger hospitals (hub) [9]. After the assessment of patient’s clinical and laboratory data, the definition of biopsy indications with the expertise in performing the procedure at the nephrology center, the prompt production of an informative histopathological report and the consequent multidisciplinary discussion are helpful to ensure the best therapeutic approach for each case. This can be achieved through the creation of appropriate collaboration agreements among the spokes and the hub for the routine processing and analysis of renal biopsy by all the needed pathology techniques (eg. LM, IF, TEM). To be eligible for such agreements, a hub should be able to maintain a TAT for reporting as short as possible, with > 80% of cases with a TAT < 5 days (at least for optical microscopy and IF) [10]. In the present experience, only 8.6% of cases had a TAT > 5 days. This system allows the nephrologist to access encrypted digital reports and slides, enhancing the continuous exchange of clinical data and critical opinions, eventually asking for second opinions [11]. In the present series, nephrologists accessed to the scanned renal biopsy after 24–48 h, leading to the real time discussion of the case with pathologists and a consequent integrated clinico-pathological report.

**Fig. 3:**
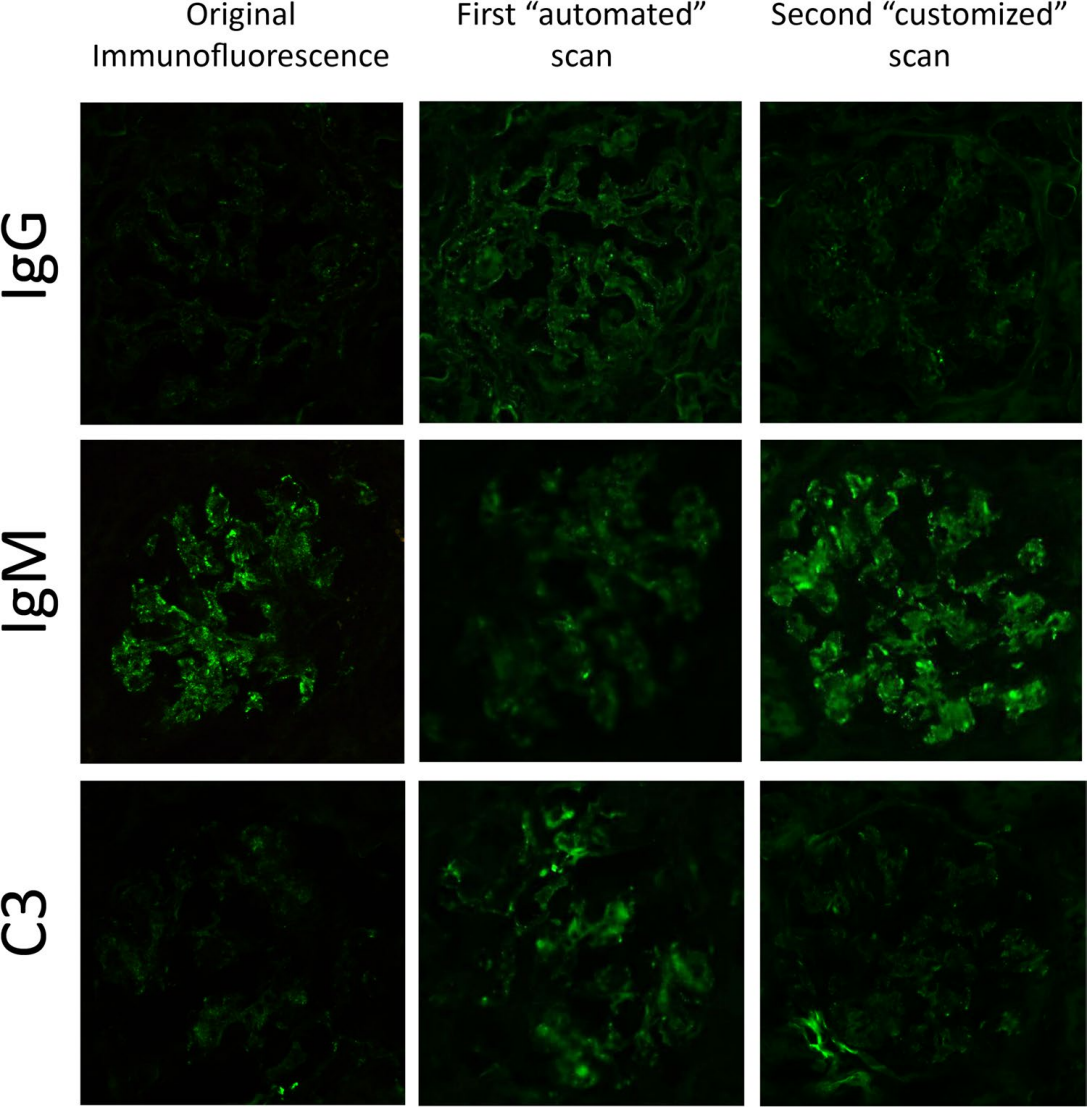
A case of mesangioproliferative glomerulonephritis in a patient with mixed connective tissue disease (MCTD). Original immunofluorescence (first column), captured with a static camera associated with the microscope, showed the presence of IgM dominant immune complexes, consistent with a MCTD-associated immune complex glomerulonephritis. The automated scan using the focus and exposition time preset by Aperio device (middle column) failed to demonstrate the prevalence of IgM antisera, showing even higher intensity for C3. Adjusting the focus and exposition time manually adopting the settings of the static camera (last column) the results were more comparable with the original

Renal biopsies shipping times from distant centers, requiring up to 24 h in some cases, could represent an issue for the central hub for the risk of inappropriate preservation of the samples, especially for the IF fresh specimens. In the absence of a dedicated and properly preserved specimen, that is still considered the gold standard, the previously proposed FFPE retrieval for either IF [7] and TEM [12] represents an invaluable alternative technique. In this experience, samples received from distant afferent centers (eg. South of Italy) and/or shipped during the weekend/holidays have been whole fixed in formalin and then processed following the appropriate protocols for IF and TEM, reducing the number of suboptimal/inadequate cases (4%, *n* = 34). Paraffin IF has been performed in a minority of biopsies (30%, *n* = 246), requiring additional ultrastructural analysis for the final diagnosis only in few cases (23%, *n* = 56). Although affected by lower sensitivity for the detection of anti-GBM disease and C3 deposits [7], its employment allows to diagnose some rarer entities [13, 14]. The digitalization of IF slides can overcome the problem of time-sensitivity due to the photobleaching phenomenon, if adequately optimized to obtain a reliable reproduction of the intensity and distribution of the original stain. This is required for some entities defined by stringent IF criteria, such as the dominant and/or co-dominant staining for specific antisera (eg. IgA and/or C3). In our experience, the careful manual setting of the most appropriate focus and exposition time can represent a reliable way of immortalizing diagnostic findings, avoiding interpretation pitfalls and diagnostic problems (Fig. 3). Digital pathology could even allow the integration of electron microscopy,always considered a static technique for the need of physical preparation and analysis by the technician/pathologist, although the possibility to digitalize the ultrastructural pictures has been demonstrated [15]. Moreover, virtual microscopy could be used for the remote assessment of thick sections adequacy as well as for the selection of the region-of-interest to analyse. The crucial role still played by TEM in renal pathology is testified by the number of cases in which it has been essential 22% (75) or informative 48% (163), as already previously reported [8]. The ultrastructural analysis is essential in podocytopathies (eg. assessing the extension of foot process effacement in MCD and FSGS) [16] as well as in the setting of rare genetic diseases (eg. determining the presence of myelin bodies in Fabry nephropathy), although the employment of paraffin retrieved TEM in cases of suspect Alport disease is limited by its unreliability in the determination of GBM thickness [17]. Electron microscopy has been informative in MN, defining the disease stage on the base of deposits re-absorption grade and detecting subendothelial/mesangial deposits in suspect secondary forms, as well as in cases of suspect mixed class lupus nephritis, with the characterization of the extent of subepithelial deposits. Finally, ultrastructural analysis has been even rarely informative in IgA nephropathy and DN or ANS, contributing to a better definition of the disease in roughly 20% of cases with inadequate/insufficient LM and IF samples or early stage diseases.

Although the initial difficulties in setting up the complex infrastructural system, the digital pathology facility can contribute to the creation of kidney biopsy registries with epidemiologic purposes. In the present study, the most frequent glomerulonephritides were represented, in order, by MN, IgA nephropathy, FSGS and MCD, both in the whole 6-years period and in the single year analysis. These data are substantially concordant with a recent study analysing the incidence of renal diseases in China [18] as compared to European countries [19, 20]. Possible reasons of these discrepancies could be different bioptic policies, geographical regions and variable availability of ancillary tools for the histological diagnosis (eg. TEM) [21].

**Fig. 4:**
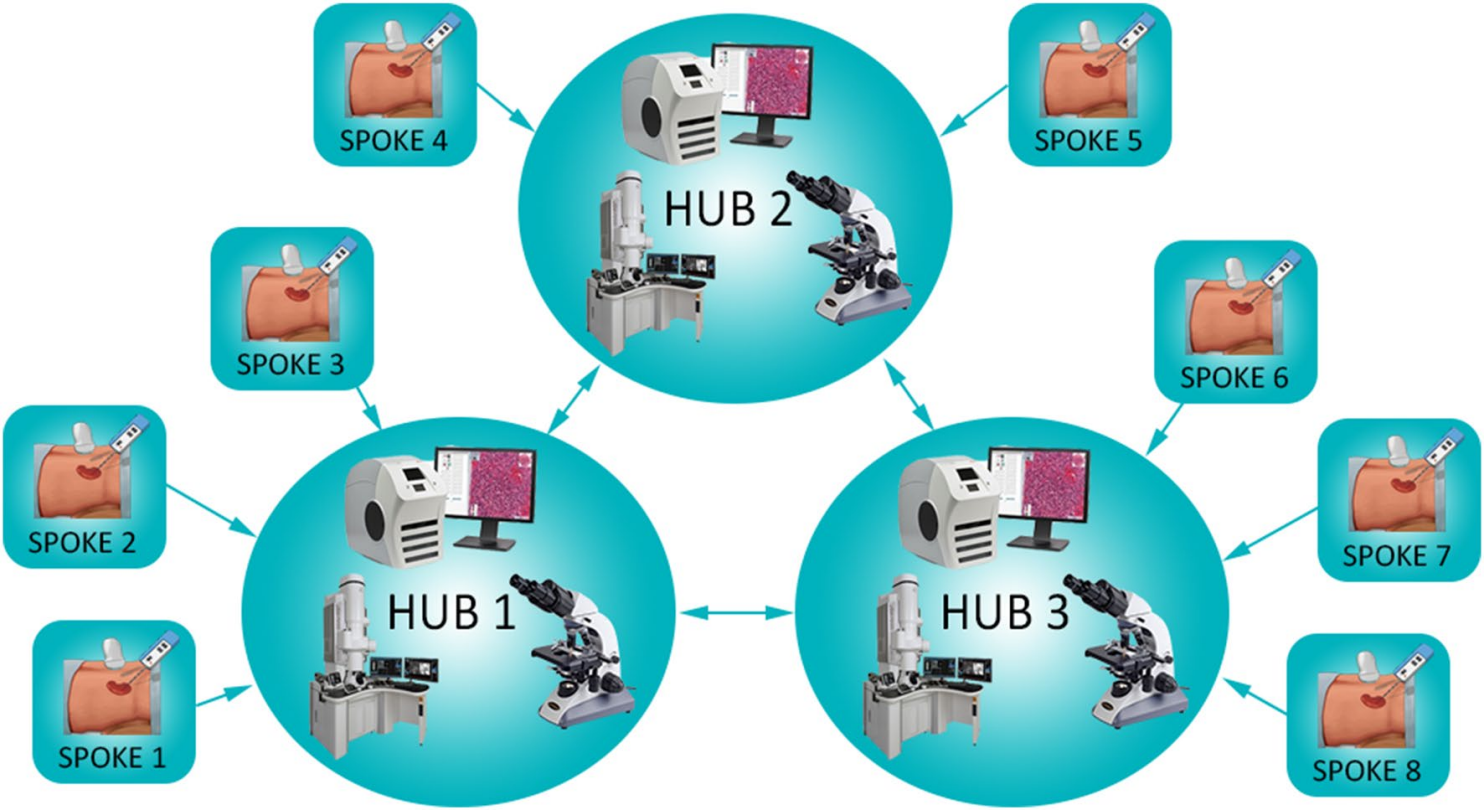
The creation of a network with spokes (nephrology centers that perform the biopsy) and hubs (big pathology centers with electron microscopy and digital pathology facilities) allows the referral clinician to rely on the expertise of specialist with a consolidated experience in the field of renal pathology. On the other hand, the hubs can communicate for second opinion and research purposes

Finally, the creation of an integrated network among spokes and hubs, facilitated by the implementation of digital pathology (Fig. 4), allows the creation of large dataset for clinical trials and research [22], allowing the employment of innovative proteomic tools [23]. The access to a digitalized database of renal biopsies can also have an educational role [24, 25], even in a telematic fashion [26], as required by the recent epidemics worldwide. The conversion to the WSI can initially raise some concerns, mainly regarding the consolidated role of traditional microscopy, often considered by far more impressive and instructive than the digital images. However, the performance of an adequate renal pathology training in specialized hubs for both pathologist and nephrologists, the implementation of adequate devices (eg. wide high resolution monitors) [28] and the adjustments of scanning procedures for IF, as demonstrated in this study, are required to exploit the potential of virtual microscopy, leading to non-inferior results as compared to the traditional counterpart [27]

## Conclusions

In the present experience we demonstrated the feasibility and sustainability of the digital switch in renal pathology routine as a possible alternative standard of care in renal pathology. The integrated workflow and the optimized employment of different pathology techniques significantly improved the diagnostic performance and reduced the turnaround-time.
